# Simeprevir with peginterferon/ribavirin for patients with hepatitis C virus genotype 1: high frequency of viral relapse in elderly patients

**DOI:** 10.1186/s40064-016-2190-9

**Published:** 2016-04-26

**Authors:** Takao Watanabe, Kouji Joko, Hirotaka Seike, Kojiro Michitaka, Norio Horiike, Yoshiyasu Kisaka, Yoshinori Tanaka, Seiji Nakanishi, Kimio Nakanishi, Takashi Nonaka, Kazuhiko Yamauchi, Morikazu Onji, Yoshinori Ohno, Yoshio Tokumoto, Masashi Hirooka, Masanori Abe, Yoichi Hiasa

**Affiliations:** Department of Gastroenterology and Metabology, Ehime University Graduate School of Medicine, Shitsukawa, Toon, Ehime 791-0295 Japan; Center for Liver-Biliary-Pancreatic Diseases, Matsuyama Red Cross Hospital, 1 Bunkyocho, Matsuyama, Ehime 790-8524 Japan; Department of Gastroenterology, Uwajima City Hospital, 1-1 Gotenmachi, Uwajima, Ehime 798-0061 Japan; Department of Gastroenterology, Ehime Prefectural Central Hospital, 83 Kasugamachi, Matsuyama, Ehime 790-0024 Japan; Department of Gastroenterology, Saiseikai Imabari Hospital, 7-1-6 Kitamura, Imabari, Ehime 799-1502 Japan; Department of Gastroenterology, Matsuyama Shimin Hospital, 2-6-5 Ootemachi, Matsuyama, Ehime 790-0067 Japan; Department of Gastroenterology, Ehime Prefectural Imabari Hospital, 4-5-5 Ishiicho, Imabari, Ehime 794-0006 Japan; Department of Internal Medicine, Shiritsu Oozu Hospital, 570 Kou Nishioozu, Oozu, Ehime 795-0013 Japan; Department of Internal Medicine, Ehime Prefectural Niihama Hospital, 3-1-1 Hongo, Niihama, Ehime 792-0042 Japan; Department of Gastroenterology, National Hospital Organization Ehime Medical Center, 366 Yokogawara, Toon, Ehime 791-0203 Japan

**Keywords:** Simeprevir (TMC435), Elderly patients, Viral relapse, Adverse events

## Abstract

**Purpose:**

The tolerability and efficacy of simeprevir in combination with peginterferon and ribavirin in patients infected with hepatitis C virus (HCV) genotype 1 under actual clinical conditions were investigated.

**Methods:**

A total of 176 patients with chronic HCV genotype 1 infection were treated with simeprevir for 12 weeks plus Peg-IFN/RBV for 24 weeks. Overall, 107 (60.7 %) patients were aged 60 years or more, and 16 (9 %) patients were aged 70 years or more. Treatment discontinuation, sustained virological response 12 (SVR12), and viral relapse were evaluated and compared between younger patients and elderly patients.

**Results:**

The rates of undetectable HCV RNA at the end of treatment were 95.8, 100 and 93.1 % in treatment-naïve, prior relapse, and prior non-responders, respectively. However, the rates of SVR12 were 82.4, 88.2 and 69.2 %, respectively. Especially in prior non-responders, viral relapse was relatively frequent. Treatment discontinuation and SVR12 were not different between patients aged <70 and ≥70 years, but viral relapse after completing treatment was significantly more frequent in patients aged ≥70 years (p = 0.012).

**Conclusions:**

In simeprevir with peginterferon and ribavirin therapy, viral relapse was relatively frequent. Especially in elderly patients, the relapse rate was high after completing treatment, instead of low frequency of discontinuation by the adverse events.

## Background

Hepatitis C virus (HCV) infection is a major public health concern. About 150 million individuals are infected worldwide, and every year, 3–4 million individuals become infected with the virus (European Association for the Study of the Liver 2011; Manns and von Hahn 2013). Japan has one of the highest rates of HCV infection worldwide, and around 2 million people are estimated to be infected, with the majority being infected with HCV genotype 1b (Chung et al. 2010). Chronic infection and liver cirrhosis with HCV are major causes of liver disease (Lavanchy 2009), and hepatocellular carcinoma is a leading cause of cancer mortality in Japan, with more than 70 % of the cases related to HCV infection (Yuen et al. 2009).

The goal of chronic HCV infection treatment is virus eradication, to prevent progression to cirrhosis and hepatocellular carcinoma. Combination therapy with peginterferon (Peg-IFN) and ribavirin (RBV) for 48–72 weeks has been standard care for HCV genotype 1 infection for many years (Editors of the Drafting Committee for Hepatitis Management Guidelines: The Japan Society of Hepatology 2013; Ghany et al. 2011), resulting in sustained virologic response (SVR) in approximately 50 % of patients (Kuboki et al. 2007; Manns et al. 2001).

The development of direct-acting antiviral agents (DAAs), including protease inhibitors (PIs), represents a major breakthrough in the treatment of chronic HCV infection. The addition of PI to Peg-IFN and RBV has markedly improved treatment outcomes in both treatment-naïve and treatment-experienced patients (Bacon et al. 2011; Hayashi et al. 2012; Jacobson et al. 2011; Kumada et al. 2012). However, first-generation HCV PIs, such as telaprevir and boceprevir, are associated with multiple daily dosing and the potential for adverse events, including anemia, rash, and renal dysfunction (Poordad et al. 2011; Zeuzem et al. 2011), leading to high rates of treatment discontinuation (Hayashi et al. 2012; McHutchison et al. 2009).

Simeprevir (TMC435) is a once-daily, oral HCV PI, with potent antiviral activity against HCV genotype 1, as well as against genotypes 2 and 4–6, although the efficacy against genotype 2 has been demonstrated only in vitro (Reesink et al. 2010; Moreno et al. 2012). In international phase 3 studies, simeprevir combined with Peg-IFN and RBV has shown good tolerability and high SVR rates in both treatment-naïve (Jacobson et al. 2014; Manns et al. 2014) and treatment-experienced patients (Forns et al. 2014; Reddy et al. 2015). In these studies, almost all treated patients were genotype 1 (1a: 41–56 %, 1b: 44–58 %), and 7.0–15.6 % of patients had liver cirrhosis. Japanese phase 3 studies of simeprevir combined with Peg-IFN and RBV have been reported in treatment-naïve patients (CONCERTO-1, 4) (Hayashi et al. 2014; Kumada et al. 2015), non-responders (CONCERTO-2), and relapsers (CONCERTO-3) to previous IFN-based therapy (Izumi et al. 2014). In these Japanese studies, almost all treated patients were genotype 1b, and no patients with liver cirrhosis were included.

However, in Japan, after these clinical trials, the tolerability and efficacy in patients under real-world conditions have not been reported. Additionally, in the CONCERTO 1–4 study, the eligible patients were younger than 70 years, even though in Japan, the percentage of elderly patients with hepatitis C virus infection is high (Tanaka et al. 2004, 2011). In this study, efficacy and treatment discontinuation for adverse events of simeprevir/Peg-IFN/RBV therapy were evaluated in actual clinical practice, and these were compared between younger patients and elderly patients.

## Patients and methods

### Study population

A total of 176 patients with chronic HCV genotype 1 infection and plasma HCV RNA of 5.0 log_10_IU/mL or more at screening were treated with simeprevir for 12 weeks plus Peg-IFN/RBV for 24 weeks at 10 hospitals belonging to the Ehime Kan-en Network (EKEN net; Ehime University Hospital, Matsuyama Red Cross Hospital, Ehime Prefectural Central Hospital, Uwajima City Hospital, Saiseikai Imabari Hospital, Matsuyama Shimin Hospital, Ehime Prefectural Imabari Hospital, Shiritsu Oozu Hospital, Ehime Prefectural Niihama Hospital, and National Hospital Organization Ehime Medical Center). The Ethics Committee of Ehime University Hospital approved the study protocol (approval ID 1411010), which conformed to the ethical guidelines of the Declaration of Helsinki amended in 2008. Written, informed consent was obtained from each patient.

Exclusion criteria included liver cirrhosis or hepatic failure, liver diseases of non-HCV etiology, co-infection with non-genotype 1 HCV, hepatitis B virus, HIV-1 or HIV-2, and any other clinically significant disease, organ transplant, or defined obvious laboratory abnormalities at screening, as well as a history of hepatocellular carcinoma within 5 years before study entry.

### Treatment administration

Simeprevir (Janssen Pharmaceutical K.K., Tokyo, Japan) 100 mg was administered orally once daily as a single capsule. Simeprevir dose adjustments were not permitted. Two kinds of Peg-IFN/RBV were administered, namely Peg-IFN α-2a (Pegasys^®^, Chugai Pharmaceutical Co. Ltd, Tokyo, Japan) and RBV (Copegus^®^, Chugai Pharmaceutical Co. Ltd) or Peg-IFN α-2b (PegIntron^®^, Merck Sharp & Dohme, Whitehouse Station, NJ, USA) and RBV (Rebetol^®^, Merck Sharp & Dohme). Peg-IFN α-2a was administered by subcutaneous injection (180 μg once weekly), and Peg-IFN α-2b was administered by subcutaneous injection (1.5 μg/kg body weight). RBV was administered as oral tablets (600–1000 mg total daily dose, depending on body weight). Dose changes, temporary interruptions, or discontinuation of Peg-IFN and RBV had to be conducted in accordance with the manufacturer’s prescribing information.

Patients stopped simeprevir if they experienced any of the following: grade 4 elevation of total bilirubin or aspartate transaminase; grade 3/4 skin events/allergic reactions; or worsening of hepatic disease. All study medications were stopped if patients experienced grade 4 adverse events or laboratory abnormalities that were not considered to be related to simeprevir specifically or were not expected toxicities of Peg-IFN/RBV or HCV infection or if patients experienced worsening of hepatic disease.

Additionally, all study medications were stopped if the following defined virologic stopping criteria were met: <2 log_10_IU/mL reduction in HCV RNA at week 12 relative to baseline, or HCV RNA levels of more than 2 log_10_IU/mL at week 12.

### Study assessments

Plasma HCV RNA was quantified at screening at baseline, and at 1, 2, 3, 4, 6, 8, 12, 24, 28 and 36 weeks using the Roche COBAS^®^ TaqMan^®^ HCV Auto assay system (Roche Molecular Diagnostics, Pleasanton, CA, USA) with a lower limit of quantification of 1.2 log_10_IU/mL.

The major efficacy end-point was the proportion of patients with undetectable HCV RNA at the end of treatment and 12 weeks after the last treatment (SVR12). Other efficacy end-points included the proportion of patients with: undetectable HCV RNA at the end of treatment (EOT); undetectable HCV-RNA at the end of treatment and 4 weeks after the last treatment (SVR4); increase of >1 log_10_IU/mL in plasma HCV RNA level from the lowest level reached or plasma HCV RNA level >2.0 log_10_IU/mL in patients whose plasma HCV RNA level had previously been <1.2 log_10_IU/mL detectable or undetectable (viral breakthrough); detectable or quantifiable plasma HCV RNA during the post-treatment follow-up period in patients who had undetectable plasma HCV RNA at the end of treatment (viral relapse); and the proportion of patients who discontinued treatment due to adverse events or virologic stopping criteria.

### Statistical analysis

Differences were evaluated using the χ^2^-test, Student’s *t* test, or Welch’s *t* test. Factors that were not normally distributed were evaluated by Welch’s *t* test. Predictors of SVR12 and viral relapse after treatment completion were evaluated using multivariate logistic regression analyses. Odds ratios (ORs) and 95 % confidence intervals were also calculated. All p values <0.05 on two-tailed testing were considered significant. Data were statistically analyzed using SPSS software ver. 18.

## Results

### Patients

This was a prospective, multicenter study. In total, 176 patients received treatment; 85 patients were treatment-naïve, and 90 patients were treatment-experienced. In treatment-experienced patients, 51 patients were prior relapsers (patients who had undetectable levels of HCV RNA at the last assessment while on IFN-based therapy and subsequent detectable levels of HCV RNA within 12 months from their last treatment), 26 patients were prior non-responders (patients who did not achieve undetectable HCV RNA on prior IFN-based therapy), and there was no prior treatment information for the remaining 12 patients. Demographic and disease characteristics at baseline are shown in Table 1. The patients’ median age was 62 years (range 28–78 years); 107 (60.7 %) patients were aged 60 years or more, and 16 (9 %) patients were aged 70 years or more. IL28B rs8099917 polymorphisms were determined in 91 patients; 69 patients were major allele TT, and 22 patients were minor allele TG/GG.

**Table 1:** Clinical and virological characteristics of patients with HCV infection

Sex (male/female)	95/81
Age (years)	62 (28–78)
AST (IU/L)	43.5 (18–217)
ALT (IU/L)	48.0 (11–225)
Total bilirubin (mg/dL)	0.8 (0.1–2.7)
Hemoglobin (g/dL)	14.5 (10.1–18.3)
White blood cells (/µL)	4905 (2100–9610)
Platelet count (×10^4^/µL)	15.3 (5.9–28.0)
HCV RNA (log copies/mL)	6.5 (5.0–7.8)
IL28B genotype (SNP8099917) (TT/non-TT)	69/22
HCV Core aa70 (wild/mutant)	38/13
HCV Core aa91 (wild/mutant)	32/19
Treatment-naïve/relapsers/non-responders/unknown	85/51/26/14
Prior therapy (IFN only/IFN + RBV/Peg-IFN/Peg IFN + RBV/teraprevir/Others)	12/7/5/49/3/12
Histological fibrosis (METAVIR score) (F0/F1/F2/F3/F4/ND)	1/28/34/30/4/79

*ALT* alanine aminotransferase, *AST* aspartate aminotransferase, *HCV* hepatitis C virus, *SNP* single nucleotide polymorphism, *Peg*-*IFN* peginterferon, *RBV* ribavirin

### Virologic response

**Fig. 1:**
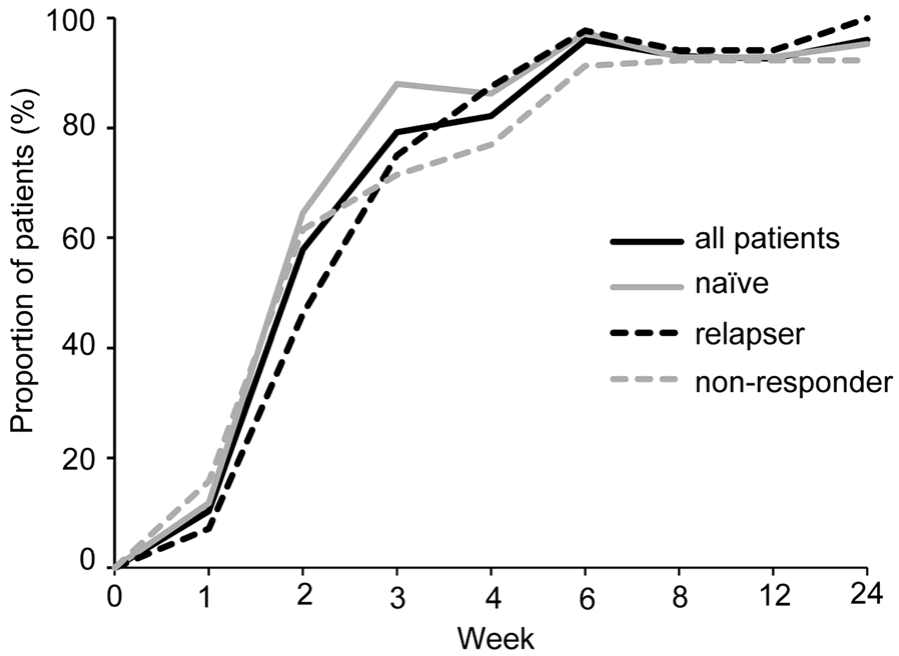
The proportion of HCV RNA negative conversion at each time point after treatment start (ITT analysis). The *black line* shows the proportion of HCV RNA negative conversion in all patients. The *gray line* shows that in treatment-naïve patients, the *black dotted line* shows that in prior relapsers, and the *gray dotted line* shows that in prior non-responders. Non-responders have slightly lower rates of HCV RNA negative conversion at each time point, but no significant differences are seen

The rates of HCV RNA negative conversion during the treatment period in patients grouped according to past treatment experience are shown in Fig. 1 (ITT analysis); most patients in all groups had achieved levels below the lower limit of quantification. Non-responders had slightly lower rates of HCV RNA negative conversion at each time point, but significant differences were not seen.

**Fig. 2:**
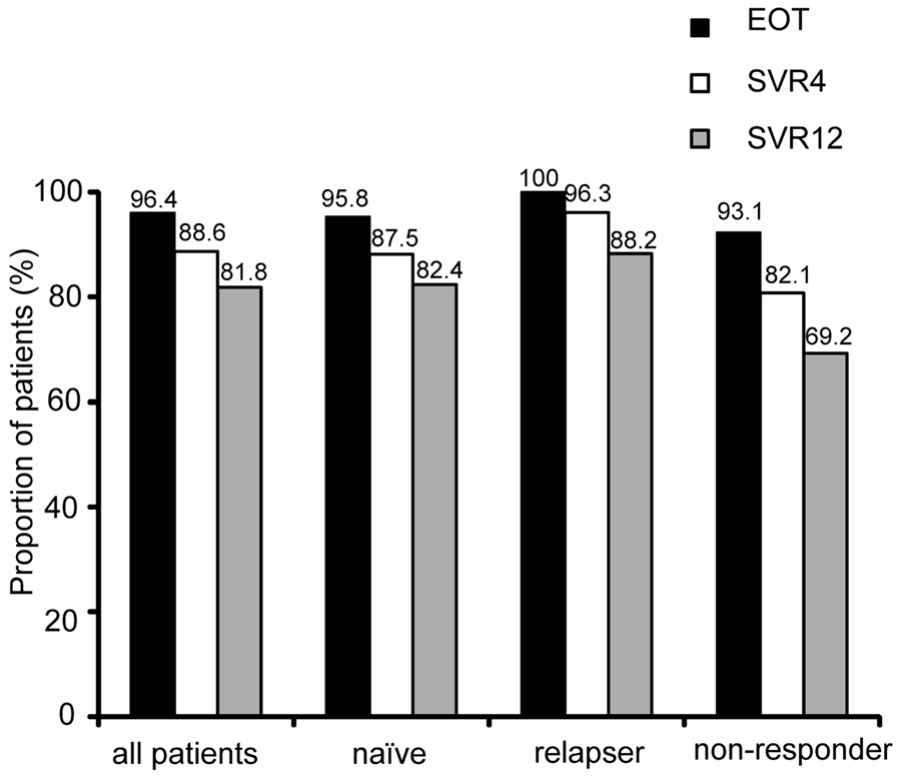
The proportions of EOT, SVR4, and SVR12 achievement in all patients, treatment-naïve patients, relapsers, and non-responders are shown. EOT, undetectable HCV RNA at the end of treatment; SVR4, undetectable HCV-RNA at the end of treatment and 4 weeks after the last treatment; SVR12, undetectable HCV-RNA at the end of treatment and 12 weeks after the last treatment

The EOT response, SVR4 response, and SVR12 response are shown in Fig. 2. In all patients, EOT, SVR4, and SVR12 responses were 96.0, 88.6 and 81.8 %, respectively. There was a relatively high frequency of viral relapse. The viral relapse rate was higher in non-responders than in treatment-naïve patients or relapsers.

### Treatment discontinuation

Treatment discontinuation was seen in 20 patients (11.3 %). The reasons for discontinuation are shown in Table 2. Viral breakthrough was seen in three patients, and two patients stopped treatment according to the virologic stopping criteria. Three of these five patients had a mutation in the HCV NS3 Protease domain at position 168 (D168V, D168E, and D168A, respectively) at the end of treatment or at viral relapse.

**Table 2:** Reasons for discontinuing treatment with simeprevir plus Peg-IFN/RBV

Reason for discontinuation	No. of patients
Viral breakthrough	3
Virological stopping criteria	2
Depression	3
Rash	1
Retinopathy	1
Anemia	3
General fatigue	5
Others	2

### Treatment discontinuation and efficacy in elderly patients

**Fig. 3:**
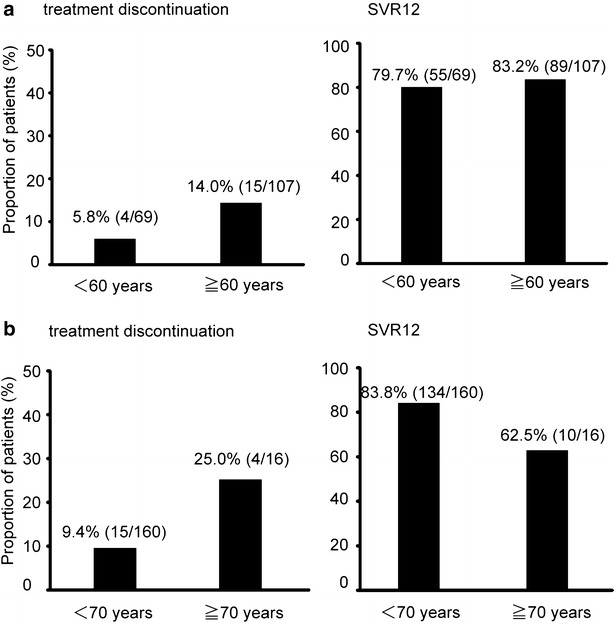
The proportions of treatment discontinuation and SVR12 achievement in patients aged 60 years or more and patients aged <60 years (**a**). The proportions of treatment discontinuation and SVR12 achievement in patients aged 70 years or more and patients aged <70 years (**b**). No significant differences are seen between younger and elderly patients

In Japan, the percentage of elderly patients with hepatitis C virus infection is high. In fact, 60.7 % of the present patients were aged 60 years or more, and 9 % of the patients were aged 70 years or more. The rates of treatment discontinuation and SVR12 achievement were compared between patients aged 60 years or more and patients aged <60 years. Similarly, the rates of treatment discontinuation and SVR12 achievement were compared between patients aged 70 years or more and patients aged <70 years. The rates of treatment discontinuation and SVR12 achievement were not significantly different between patients aged 60 years or more and patients aged <60 years (p = 0.225, p = 0.556, respectively) (Fig. 3a). Similarly, the rates of treatment discontinuation and SVR12 achievement were not significantly different between patients aged 70 years or more and patients aged <70 years (p = 0.090, p = 0.081, respectively) (Fig. 3b).

**Fig. 4:**
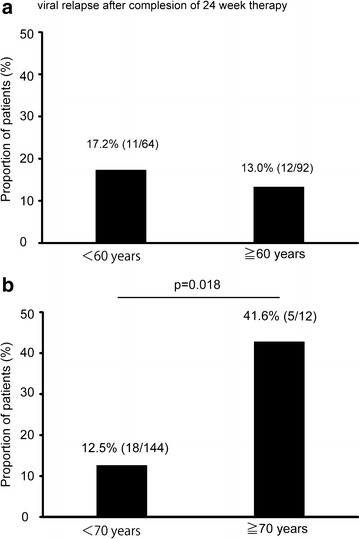
The proportions of viral relapse after 24-week therapy completion. There is no significant difference between patients aged 60 years or more and patients aged <60 years (**a**). However, viral relapse is more frequent in patients aged 70 years or more than in patients aged <70 years (**b**) (41.6 vs 12.5 %, p = 0.018)

As mentioned above, there was a relatively high frequency of viral relapse after completion of 24 weeks therapy. Thus, the rates of viral relapse after completion of 24 weeks therapy were also compared between patients aged 60 years or more and patients aged <60 years, and between patients aged 70 years or more and patients aged <70 years. The rates of viral relapse after treatment completion were not significantly different between patients aged 60 years or more and patients aged <60 years (p = 0.49) (Fig. 4a). However, viral relapse after treatment completion was significantly more frequent in patients aged 70 years or more than in those aged <70 years (41.6 vs 12.5 %, p = 0.018) (Fig. 4b).

### Predictors of SVR12 achievement

Factors that might contribute to SVR achievement were evaluated (Table 3). Potential predictive factors associated with SVR12 included age (70 years or more/<70 years), sex, ALT, platelet count, AFP, HCV-RNA, IL28B rs8099917 polymorphisms (TT/non-TT), HCV Core aa70 (wild/mutant), HCV Core aa91 (wild/mutant), treatment-naïve/treatment-experienced, non-responders to past treatment/others, Peg-IFN adherence, and RBV adherence. HCV NS3 Q80 K status, which has been reported as a predictor of simeprevir failure, was tested in 38 patients, and only 1 (2.6 %) patient had Q80 K; thus, this was not evaluated as a predictive factor. It was found that the presence of IL28B rs8099917 polymorphisms was the only predictive factor (p = 0.02). Platelet count, HCV core aa70, and non-responders tended to be associated with SVR12 achievement, but they were not significant. Multivariate analysis was performed using the factors whose p values were <0.1 on univariate analyses (age 70 years or more/<70 years, platelet count, HCV-RNA, IL28B polymorphisms, HCV core aa 70, non-responders to past treatment/others). Only IL28B polymorphism (TT vs non-TT, odds ratio: 0.145, p = 0.029) was identified as a significant factor (Table 4).

**Table 3:** Factors associated with SVR12 achievement

	SVR12	Non-SVR12	p value
Sex (male/female)	82/62	13/19	0.11
Age 70 years or more/<70 years	134/10	26/6	0.08
ALT (U/L)	62.5 ± 45.9	64.9 ± 36.4	0.81
Platelet count (×10^4^/µL)	15.8 ± 4.7	14.1 ± 4.9	0.08
AFP (ng/mL)	12.3 ± 34.4	16.0 ± 19.4	0.56
HCV-RNA (log copies/mL)	6.4 ± 0.68	6.6 ± 0.58	0.10
IL28B (TT/non-TT)	61/14	8/8	0.02
HCV Core aa70 (wild/mutant)	35/9	3/4	0.06
HCV Core aa91 (wild/mutant)	26/17	6/2	0.69
Treatment naïve/re-treatment	70/73	15/17	0.84
Non-responders/others	18/115	8/21	0.09
Peg-IFN adherence (%)	88.6 ± 22.4	78.6 ± 39.1	0.18
RBV adherence (%)	82.3 ± 22.4	76.7 ± 45.1	0.50
Histological fibrosis (F0-2/F3-4)	55/28	8/6	0.55

Data are expressed as mean ± standard deviation

*SVR* sustained virological response, *ALT* alanine aminotransferase, *AFP* α-fetoprotein, *HCV* hepatitis C virus, *Peg*-*IFN* peginterferon, *RBV* ribavirin

**Table 4:** Independent factors associated with SVR12 achievement on multiple logistic regression analysis

	Odds ratio	95 % CI	p value
IL28B (TT/non-TT)	0.145	0.026-0.822	0.029

*CI* confidence interval

### Predictors of viral relapse after treatment completion

In simeprevir/Peg-IFN/RBV therapy, since viral relapse after completion of 24 weeks of therapy was frequent, an attempt was made to identify the predictors of viral relapse after treatment completion (Table 5). Potential predictive factors associated with SVR12 included age (age 70 years or more/<70 years), sex, ALT, platelet count, AFP, HCV-RNA, IL28B rs8099917 polymorphisms (TT/non-TT), HCV Core aa70 (wild/mutant), HCV Core aa91 (wild/mutant), treatment-naïve/treatment-experienced, non-responders to past treatment/others, Peg-IFN adherence, and RBV adherence. Age 70 years or more, IL28B rs8099917 polymorphisms, and platelet count were identified as predictive factors (p = 0.018, 0.012, 0.023, respectively). Multivariate analysis was conducted using the factors whose p values were <0.1 on univariate analyses (age 70 years or more/<70 years, platelet count, IL28B polymorphisms, non-responders to past treatment/others). Age (age 70 years or more vs <70 years, odds ratio: 15.1, p = 0.037) and IL 28B polymorphisms (TT vs non-TT, odds ratio: 6.29, p = 0.022) were identified as significant factors (Table 6). The factor age 70 years or more had the highest odds ratio for predicting viral relapse after treatment completion.

**Table 5:** Factors associated with viral relapse after treatment completion

	Viral relapse	SVR12	p value
Sex (male/female)	9/14	73/60	0.18
Age 70 years or more/<70 years	5/18	7/126	0.018
ALT (U/L)	62.6 ± 38.0	63.6 ± 46.9	0.92
Platelet count (×10^4^/µL)	13.5 ± 4.5	15.9 ± 4.6	0.023
AFP (ng/mL)	16.6 ± 21.8	12.3 ± 35.4	0.58
HCV-RNA (log copies/mL)	6.6 ± 0.57	6.4 ± 0.69	0.14
IL28B (TT/non-TT)	4/6	57/14	0.012
HCV Core aa70 (wild/mutant)	1/2	33/9	0.14
HCV Core aa91 (wild/mutant)	3/1	24/17	0.64
Treatment naïve/re-treatment	10/13	66/66	0.65
Non-responders/others	6/17	16/106	0.10
Peg-IFN adherence (%)	88.6 ± 19.5	91.8 ± 18.6	0.46
RBV adherence (%)	90.6 ± 43.9	84.8 ± 20.7	0.33
Histological fibrosis (F0-2/F3-4)	7/6	52/25	0.35

Data are expressed as mean ± standard deviation

*SVR* sustained virological response, *ALT* alanine aminotransferase, *AFP* α-fetoprotein, *HCV* hepatitis C virus, *Peg*-*IFN* peginterferon, *RBV* ribavirin

**Table 6:** Independent factors associated with viral relapse after treatment completion on multiple logistic regression analysis

	Odds ratio	95 % CI	p value
Age 70 years or more/<70 years	15.1	1.18–194	0.037
IL28B (TT/non-TT)	6.29	1.29–30.5	0.022

*CI* confidence interval

## Discussion

In patients treated with just Peg-IFN and ribavirin, IL28B genotype (non-TT) and advanced fibrosis are associated with a low rate of SVR (Tanaka et al. 2009; Manns et al. 2001; Fried et al. 2002). In an international study of simeprevir and Peg-IFN/Ribavirin, simeprevir treatment also resulted in higher SVR12 in patients with advanced fibrosis than just Peg-IFN and ribavirin (Jacobson et al. 2014; Manns et al. 2014; Forns et al. 2014). In the present study, platelet count and histological fibrosis did not affect the rate of SVR12. In these international studies, since the difference in SVR12 according to IL28B polymorphism was substantially smaller than in the placebo group, the correlation between IL28B genotype and efficacy of simeprevir was smaller than for just Peg-IFN/ribavirin therapy. In a Japanese study, the results of the subgroup analysis by IL28B polymorphism also showed that there were no significant differences in efficacy according to this genotype (Hayashi et al. 2014; Kumada et al. 2015; Izumi et al. 2014). On the other hand, in the present study, only IL28B genotype was a factor affecting SVR12. Though in the past clinical studies of simeprevir therapy the median age of patients was in the forties or fifties, that of the present patients was 62 years, and patients over 70 years were also included, which would be the reason for the difference in the effect on treatment efficacy between the present results and those of past studies. In Japanese actual clinical practice, the proportion of elderly patients is high, so the present results should be taken into account.

The present study showed that, especially in prior non-responders, there was a relatively high frequency of viral relapse (p = 0.060 compared with prior relapsers). This result agreed with past studies; for example, in CONCERT-2 (prior non-responders), viral relapse was seen in 38.6 % compared to 8.2 % in CONCERT-3 (prior relapsers). In the past reports, the factors involved in viral relapse after treatment completion were not analyzed; therefore, these factors were examined in the present study. It was found that low platelet count and IL28B SNP8099917 non-TT genotype were the significant risk factors for viral relapse. Moreover, patients aged over 70 years had a significantly higher frequency of viral relapse after treatment completion. In the present study, the number of patients aged over 70 years was small, so the result must be viewed in that light. However, multivariate analysis indicated that age over 70 years was an independent contributing factor to viral relapse after treatment completion. These data indicate that, for suppression of viral relapse, the treatment effect of Peg-IFN/ribavirin is important, because hepatic fibrosis, IL28B genotype, and age have previously been reported as factors affecting SVR in Peg-IFN/ribavirin therapy (Tanaka et al. 2009; Manns et al. 2001; Fried et al. 2002; Dienstag and McHutchison 2006).

Some mutations, such as D168E/T/V, Q80R/K, and R155K, have been previously described in HCV genotype 1b after exposure to simeprevir in vitro and in clinical studies (Hayashi et al. 2014; Lenz et al. 2010; Fried et al. 2013). Additionally, the majority of patients with viral breakthrough or relapse had emerging mutations in the HCV NS3 protease domain. These mutations were mostly at position 168 (Lenz et al. 2010). In the present patients at treatment start, only one of 40 tested patients had a position 80 mutant, and all 40 tested patients were wild type at position 168 of the HCV NS3 protease domain. In the present study, viral breakthrough was seen in three patients, and two patients stopped treatment according to virologic stopping criteria. Three of these four patients had mutations in the HCV NS3 Protease domain at position 168 (D168V, D168E, D168A, respectively) at the end of treatment or viral relapse. Unfortunately, these mutations could not be examined in viral relapse patients.

In studies of first-generation protease inhibitors, rates of treatment discontinuation due to severe adverse events have been reported to be 10–20 % (Bacon et al. 2011; Hayashi et al. 2012; Jacobson et al. 2011; Kumada et al. 2012; Poordad et al. 2011). However, in simeprevir with Peg-IFN/ribavirin therapy, treatment discontinuations were much less frequent, at 3–5 % (Hayashi et al. 2014; Kumada et al. 2015; Izumi et al. 2014). Manns et al. (2015) reported a pooled safety analysis from international phase IIb and III studies. In this report, most adverse events were grade 1/2, and most grade 3/4 adverse events occurred in <5.0 % of patients. Moreover, tolerability did not vary with histological hepatic fibrosis. In the present study, the rate of treatment discontinuation was not different between patients aged <60 and ≥60 years, and even between those aged <70 and ≥70 years. These results mean that simeprevir therapy is well tolerated even in the actual clinical setting.

The combination of simeprevir and other direct-acting antivirals such as sofosbuvir as an HCV NS5B inhibitor is expected to be the treatment option for patients with chronic HCV genotype 1 infection by providing interferon-free treatment (Schinazi et al. 2014). Efficacy of the interferon-free combination of simeprevir and sofosbuvir, with or without ribavirin, in patients with HCV genotype 1 infection was reported to be more than 90 % (SVR12 rate) (Lawitz et al. 2014). Moreover, although direct-acting antiviral combinations in an interferon-free regimen would be main stream in the future, Peg-IFN and ribavirin-based treatment could remain the standard of care in parts of the world in which the very high treatment costs of direct-acting antiviral interferon-free regimens would be prohibitive. Then, the present data of simeprevir with Peg-IFN and ribavirin in the actual clinical setting would be valuable.

In conclusion, in simeprevir with peginterferon and ribavirin therapy, both efficacy and tolerability are good, even in elderly patients, but viral relapse after completing treatment was high in patients aged over 70 years. Patients <70 years old and those discontinuing previous DAAs would be suitable for this treatment.
